# Phononic integrated circuitry and spin–orbit interaction of phonons

**DOI:** 10.1038/s41467-019-10852-3

**Published:** 2019-06-21

**Authors:** Wei Fu, Zhen Shen, Yuntao Xu, Chang-Ling Zou, Risheng Cheng, Xu Han, Hong X. Tang

**Affiliations:** Department of Electrical Engineering, Yale University, New Haven, CT 06511 USA

**Keywords:** Electrical and electronic engineering, Electronics, photonics and device physics, Acoustics

## Abstract

High-index-contrast optical waveguides are crucial for the development of photonic integrated circuits with complex functionalities. Despite many similarities between optical and acoustic waves, high-acoustic-index-contrast phononic waveguides remain elusive, preventing intricate manipulation of phonons on par with its photonic counterpart. Here, we present the realization of such phononic waveguides and the formation of phononic integrated circuits through exploiting a gallium-nitride-on-sapphire platform, which provides strong confinement and control of phonons. By demonstrating key building blocks analogous to photonic circuit components, we establish the functionality and scalability of the phononic circuits. Moreover, the unidirectional excitation of propagating phononic modes allows the exploration of unconventional spin–orbit interaction of phonons in this circuit platform, which opens up the possibility of novel applications such as acoustic gyroscopic and non-reciprocal devices. Such phononic integrated circuits could provide an invaluable resource for both classical and quantum information processing.

## Abstract (web summary)

Developing planar phononic circuits analogous to photonic circuits are of interest to provide scalable advantages and complex manipulation of phonons. Here, the authors realize a phononic integrated circuit with a Gallium Nitride-on-sapphire platform, which provides strong confinement and control of phonons.

## Introduction

Phononics—the study of vibrating structure—has become an emerging research field, owing to recent progresses in micro/nano-fabrication and investigation of phononic wave dynamics, phononic crystals, and acoustic metamaterials^1–11^. The engineered phononic structures not only provide stronger phonon interaction with light and matter by reducing the device footprint, but also allow phonon reservoir engineering^12,13^ to increase phonon lifetime, boosting the performance of phononic devices to an unprecedented level^14^. Recent experiments of quantum acoustics have pushed the study of matter-phonon and photon–phonon interactions to the single-quantum level, making possible phononics-based hybrid quantum systems and quantum memories^15–22^. Compared to its photonic and electronic counterparts, phononic devices have the desired property that the GHz frequency range corresponds to wavelengths on the order of *m*, which uniquely bridges frequency and wavelength gaps between optical and electrical circuits^23–25^.

Despite the efforts to develop novel phononic structures in micro/nano-electromechanics and optomechanics^26–37^, scalable phononic integrated circuits (PnIC) remain largely unexplored compared to their electric and photonic counterparts. A critical step towards PnICs with complex functionalities is the implementation and integration of key building blocks—phononic waveguides, multiport phononic structures, evanescently coupled phononic resonators, and scalable input/output couplers—on a single chip. Recent experiments on hybrid system employing phononic wires—the core of a PnIC–are encouraging^4–11^, however, challenges remain for each of these system to form a more complex circuit.

**Fig. 1:**
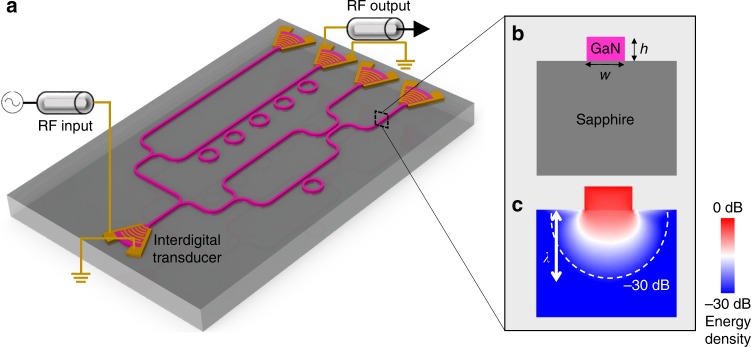
Phononic integrated circuit. **a** A notional schematic of a phononic circuit. Input radio-frequency (RF) photons are first converted into phonons by an interdigital transducer (IDT) through the piezoelectric effect of the top gallium nitride (GaN) epi-layer. The phonons are confined, routed through phononic circuit and received by output IDTs. **b** A schematic cross-section of GaN-on-sapphire strip waveguide with thickness *h* and width *w*. **c** Simulated cross-sectional acoustic energy distribution of a Rayleigh-like mode shown in logarithmic scale, indicating confinement of acoustic wave in the waveguide. The white dashed line marks where the energy density drops to 10^−3^, compared with the highest energy density

In this article, we present an experimental demonstration of a PnIC, as an analog to the photonic integrated circuit (PIC). A notional schematic of the PnIC chip is shown in Fig. 1a. An incoming radio-frequency (RF) signal from a transmission line is sent to an interdigital transducer (IDT), which converts RF photons to phonons and vice versa. The generated phonons are then confined, guided, and routed on the top layer of the chip by the phononic waveguides and go through processing circuits such as an array of ring resonators. Finally, the transmitted phonons are collected and converted back to RF photons by the output IDTs. The system described above can be summarized to three critical components: (1) scalable input and output ports; (2) phononic circuitry to confine, guide, and route phonons; (3) ring resonators to store, enhance, and modulate phonons. Interestingly, with such a PnIC, the highly confined, unidirectionally excited whispering-gallery modes carry both orbital angular moment and spin. We demonstrate, for the first time, the spin–orbit interaction (SOI) of propagating phonons. The phononic SOI brings new concept in designing of phononic system at sub-wavelength scale, opens possibilities of chiral phonon–matter interaction and non-reciprocal phononic devices^38^.

## Results

### Phononic strip waveguide

At the core of the proposed circuits is low loss, high-acoustic-index-contrast, single-mode phononic waveguides for routing phonons between localized phononic components such as phononic resonators. For short-distance acoustic wave propagation, it is possible to create such waveguides from suspended structures using surface micromachining techniques, such as phononic crystal waveguides supported in a suspended membrane^4–8^ and suspended wires supported by localized tethers^9–11^. However, due to the three-dimensional nature of these structures, it is a major challenge to freely lay out waveguide patterns with a complexity similar to that of photonic or electrical wires on a planar chip. Here, we present an alternative phononic architecture with phononic waveguides that harness acoustic velocity mismatch^39^, as shown in Fig. 1b, in a way similar to high-index contrast photonic waveguides in PIC.

We survey a number of material platforms for building acoustic waveguide structures considering the following criteria: (1) the top layer has an acoustic velocity lower than that of the substrate for a strong confinement of acoustic waves; (2) the top layer is piezoelectric to allow efficient coupling to RF input/output fields; (3) the material selected is compatible with standard wafer-scale semiconductor fabrication processing. Table 1 summarizes commonly utilized piezoelectric materials and their substrates. Gallium nitride (GaN)-on-sapphire (GNOS)^40^ stands out as a unique material platform that meets all these requirements. The ever-growing demand for light-emitting devices and high-power electronics has led GaN to become a mature semiconductor material. Moreover, the GNOS platform is compatible with standard semiconductor fabrication procedures (see Methods and Supplementary Note 2 for details about simulations and fabrications).

**Table 1:** Survey of acoustic waveguide and substrate materials

Material	Longitudinal wave speed (m s^−1^)	Transverse wave speed (m s^−1^)	Piezoelectricity
GaN^54^	7350	4578	Yes
AlN^54^	10,169	6369	Yes
Quartz^55^	5700	3158	Yes
Sapphire^56^	10,658	5796	No
Silicon^57^	8433	5843	No
Silica^55^	5800	3700	No
Diamond^58^	18,000	12000	No

The simulation results by the finite-element method in Fig. 1c shows cross-sectional elastic energy distribution, where the phonon field is mostly confined in the GaN waveguide and decays exponentially with increasing depth into the substrate. Without loss of generality, we have taken Rayleigh-like mode (to be introduced in the following section) as an example. As indicated by the white dashed line, elastic energy decays to 10^−3^ compared with the highest energy density within a distance of only one wavelength.

**Fig. 2:**
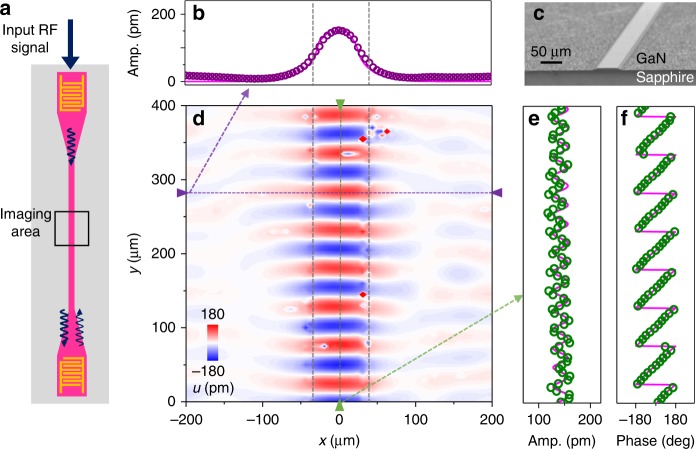
Characterization of phononic waveguide and identification of a traveling Rayleigh-like mode. **a** The measurement scheme. **c** An scanning electron microscope (SEM) image of a phononic waveguide. **b**, **d**–**f**, The out-of-plane (*z*-direction) displacement of a traveling Rayleigh-like mode. The data are measured by sending RF signal at 102 MHz into the IDT and scanning the vibrometer focal point in an area of 400 μm × 400 μm as illustrated in **a**. The reconstructed image of instant displacement is shown in **d**. The amplitude or phase at cross-sections of the two-dimensional scan are plotted in **b**, **e**, and **f** and taken at correspondingly colored lines in **d**. The magenta line in **b** represents the simulated displacement amplitude cross the waveguide, and the magenta lines in **e** and **f** are fitted to traveling acoustic wave with weak reflection (10% reflection)

In the experiment, we first consider a phononic waveguide device (Fig. 2c) optimized to support acoustic waves with wavelength around 50 μm (frequency around 100 MHz). The waveguide is 5 μm tall and 50 μm wide, which provides strong confinement while suppressing higher-order modes. As illustrated by the schematic in Fig. 2a, the phonon mode is actuated by an IDT at the end of the phononic waveguide. The IDT is composed of two comb electrodes deposited on the surface of GNOS, with parallel fingers interdigitated to provide a periodically distributed electric field. The acoustic wave is actuated by the electric field through piezoelectric effect, and the maximum coupling efficiency is achieved when the acoustic wavelength matches the period of the IDT fingers. In our experiments, the efficiency of a single IDT is around −35 dB.

To experimentally characterize the phononic devices, we employ a home-built vibrometer to map out both the amplitude and phase of the phonon modes’ out-of-plane displacement *u* (see Methods and Supplementary Note 2 for details of the vibrometer). As indicated by the black box in the measurement schematic (Fig. 2a), we scan a chip area of 400 μm × 400 μm with input RF excitation frequency at 102 MHz for the maximum IDT efficiency. The reconstructed out-of-plane displacement of the acoustic waveguide mode is shown in Fig. 2d. Good agreement between the measured (purple dots) and simulated (red line) amplitude cross the waveguide in Fig. 2b confirms that the acoustic wave is indeed confined. The amplitude along the center of the waveguide is plotted in Fig. 2e; no obvious decay is observed within the scanning area. The slight periodic oscillation of the amplitude is due to the reflection from the output IDT, which can be fitted by taking into account a reflection factor of 10% (red line in Fig. 2e). Figure 2f shows the phase distribution, which linearly increases along the propagation direction, revealing the itinerant nature of phonons in the waveguide.

### Phononic ring resonator

**Fig. 3:**
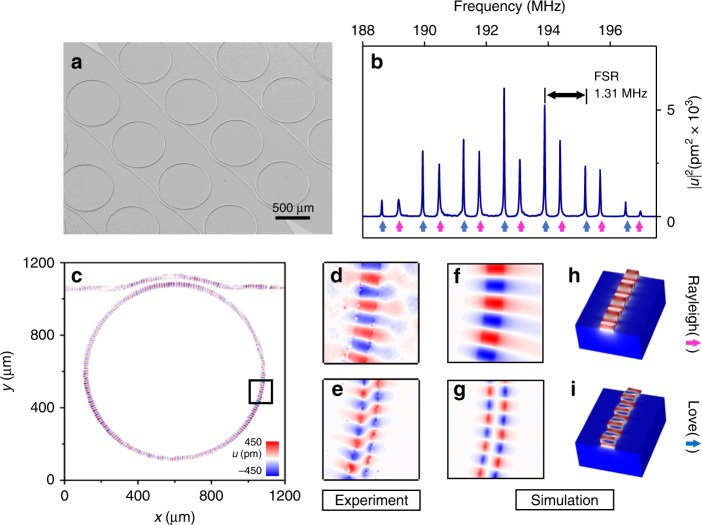
Phononic ring resonator and mode identification. **a** An SEM image of arrays of phononic ring resonators. **b** A typical spectrum of the phononic ring resonator measured by the vibrometer with the focal point adjusted to the ring. Two sets of modes are observed, corresponding to Rayleigh-like and Love-like mode, marked with magenta and blue arrows, respectively. **c** The vibrometer image of a Rayleigh-like mode in a waveguide coupled ring resonator (only the displacement on the ring is presented). **d**, **e** The vibrometer images of the Rayleigh-like and Love-like modes. **f**, **g** Simulated *z*-direction displacement profile of Rayleigh-like and Love-like modes, in excellent agreement with the experimental results. **h**, **i** Simulated 3-d mode profiles of Rayleigh-like and Love-like mode, respectively

Ring resonators are essential for PnIC and can enable narrow band signal filtrations, phonon bufferings and memories, as well as enhancement of phonon–matter/photon interaction^41–43^. For unidirectional actuation of the phonon modes, directional coupler structures are designed, simulated (see Supplementary Note 1), and here experimentally demonstrated in the form of wrap-around coupler. Figure 3a presents an SEM image of phononic ring resonators coupled with a wrap-around bus phononic waveguide, which is fabricated from a 5-μm-thick GaN film to support phonon modes at a wavelength of around 25 μm. By adjusting the vibrometer laser focal point on the ring and simultaneously sweeping the RF input frequency, we acquire an intracavity displacement spectrum as shown in Fig. 3b. Similar to photonic rings that support both transverse electric (TE) and transverse magnetic (TM) modes^44^, phononic rings support phonon modes with two orthogonal polarizations: Rayleigh-like modes (labeled by magenta arrows) and Love-like modes (labeled by blue arrows). Both families of modes show a free spectral range (FSR) of around 1.31 MHz, corresponding to a group velocity (*v*_g_) of around 4180 μm s^−1^.

To further characterize the modes and determine their polarization, we actuate the ring at different resonant frequencies and image the resonance mode profiles using the vibrometer. Figure 3c shows a displacement profile of the ring excited at 193.085 MHz, where a Rayleigh-like resonance can be clearly identified with an azimuthal number *m* = 115. Zoomed-in views of the Rayleigh-like mode and a Love-like mode excited at 192.575 MHz are shown in Fig. 3d, e, respectively, in exceptional agreement with the simulation results (Fig. 3f, g). The three-dimensional simulated mode profiles of both modes are presented in Fig. 3h, i: the Rayleigh-like mode is dominated by the out-of-plane displacement, while the Love-like mode is dominated by the in-plane displacement. Moreover, we study the intrinsic and the external decay rates of the phononic rings with different radii and coupling gaps. The highest *Q* of 2.5 × 10^4^ is obtained at room temperature, which is significantly improved to 3.2 × 10^5^ at 50 mK measured in a dilution refrigerator. We can further estimate the phonon loss rate per unit length by the *Q*s: the loss rate is $\alpha = \frac{\omega }{{Qv_{\mathrm{g}}}} = 0.5\,{\mathrm{dB}}$ per cm at room temperature and 0.03 dB per cm at 50 mK (see more details of decay rate study in the Supplementary Note 2).

### Spin–orbit interactions of phonons

**Fig. 4:**
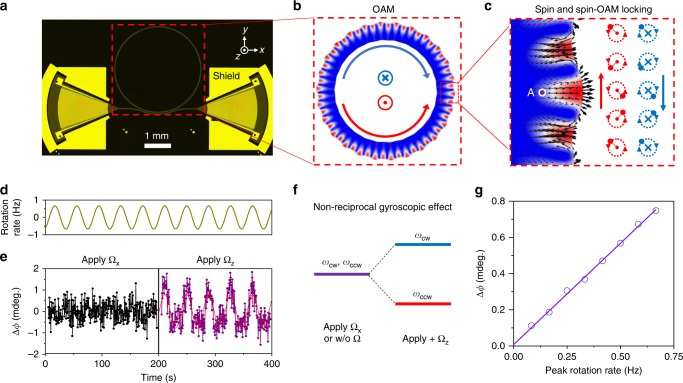
Phononic spin–orbit interaction in a ring resonator. **a** Device under test. The pair of IDTs function as the input and output of the circuit and address the clockwise (CW) and counter-clockwise (CCW) love-like mode simultaneously. Focused IDTs and shield structure are used to improve signal to noise ratio. **b** Orbital angular momentum (OAM) carried by Love-like mode. **c** Left: Zoomed in mode profile of Love-like mode, with displacements indicated by the black arrows. We observe circular trajectory of displacement. Right: Spin-momentum locking—CW and CCW propagating phonons possess spins of opposite direction. The direction of spin of point A (the purple dot in the left panel) is indicated by either cross (right-hand) or dot (left-hand). Dash line indicates the trajectory of displacement (solid dot). **d** Phase response difference between CW and CCW modes, when the device is subject to in-plane and out-of-plane rotation. The comparison between Δ*ϕ* measured with rotation of different direction is a clear signature of SOI. **e** Applying an out-of-plane CCW rotation to the system, CW and CCW Modes have resonant frequency shift towards the opposite direction, due to the opposite chiralities that they possess. **f** Phase response measured at various peak rotation rate

Similar to optical whispering-gallery modes^45^, phonon modes traveling in a ring resonator carry orbital angular momentum (OAM) ***L***, as illustrated in Fig. 4b. Depending on the propagation directions of the mode (clockwise (CW) or counter-clockwise (CCW)), ***L*** can point along negative or positive *z*-axis, respectively. At the same time, traveling Love-like modes also carry spin: the sidewall surface and the bending-induced asymmetry cause hybridization between transverse and longitudinal displacements, resulting in a circular-like trajectory, as illustrated in the enlarged simulation plot in Fig. 4c. We define the left-hand (CCW) and the right-hand (CW) circular motions as two ‘eigenstates’’ of the spin, and introduce chirality ***χ*** to characterize the collective spin motion of all particles in the ring (see Supplementary Note 1 for detailed definition of OAM and chirality of phonon modes). Moreover, the spin and the OAM of phonons are coupled due to the sub-wavelength scale confinement. Figure 4c showcases a point (indicated by purple dot) possess right-hand (left-hand) spin when phonons propagate in CW (CCW) direction. As a result of this spin–orbit interaction (SOI), phonons circulating in a ring resonator possess chiralities that are in the same direction with the OAM, also known as spin–orbit locking^46^.

In order to experimentally demonstrate the SOI of phonons, we harness the gyroscopic effect to induce a spin-dependent velocity change of the acoustic wave. The spin-dependent gyroscopic effect of phonon originates from the Coriolis forces on particles in the presence of rotation ***F*** =  −2 m**Ω** × ***v***, where **Ω** is the rotation with respect to the inertial frame of reference and ***v*** is the velocity of the particles. Particles spinning in parallel or anti-parallel direction of the rotation experience centrifugal or centripetal Coriolis force, respectively. For Love-like modes, the Coriolis force causes resonant frequency shift depending on the chirality of the mode *ω* = *ω*_0 _− ***χ*** · **Ω**^47^ (see Supplementary Note 1 for details). Therefore, by observing the OAM-dependent frequency shift (Fig. 4e) under a rotation along ***χ*** direction, we are able to confirm that the direction of chirality is the same with that of the OAM, manifesting the spin–orbit locking and the SOI of phonons.

In our experiment, we use a phononic ring device shown in Fig. 4a to test the OAM-dependent response of phononic modes. The device incorporates a pair of carefully engineered IDTs to unidirectionally actuate and detect the CW and the CCW modes simultaneously. The device is mounted on a rotating stage and a sinusoidally varying rotation is applied to the system with a peak rate at 0.667 Hz (the upper panel of Fig. 4d). To monitor the resonant frequency shift under rotation, we send to the device CW and CCW RF input signals at the original resonant frequency and record the phase change of the output signals. The difference in phase change between CW and CCW signals Δ*ϕ* = *ϕ*_cw_(Ω(*t*)) − *ϕ*_ccw_(Ω(*t*)) is plotted in the lower panel of Fig. 4d. Both in-plane (Ω_x_) and out-of-plane (Ω_z_) rotations are measured, with the system drifts calibrated out (see Supplementary Note 2 for details). When the device is subject to Ω_x_ rotation, we do not observe significant phase response, confirming |*χ*_x_| = 0 (due to the symmetry of the ring). When the device is subject to Ω_z_ rotation, however, we observe a phase response possesses the same period with the modulation of the input rotation, which is a clear signature of the OAM-dependent frequency shift. We further measure the phase response at varied peak rotation rates. By fitting the sinusoidal output signal, we acquire phase responses at rotation rate varying from 0.083 Hz to 0.667 Hz (Fig. 4f). The linearly increasing phase response to the rotation is in good agreement with theory. With the slope of the phase response signal calibrated against the phase slope of the resonance, we are able to calculate the chirality of the Love-like mode, |*χ*_z_| = 0.09, which is in a reasonable agreement with the simulation result of |*χ*_z_| = 0.12. The discrepancy might be attributed to the inaccurate material parameters used in the simulation and the mode perturbation caused by the anisotropic substrate and the coupling bus waveguide. Therefore, we confirm the spin–orbit locking and the SOI of phonons. It is worth noting that the SOI and non-reciprocity of optical photons have been observed and studied^46,48–52^, and this non-reciprocal frequency shift of phonon mode is in particular analogous to Fizeau drag of photons, which has been recently demonstrated in a spinning whispering-gallery resonator^52^. Owing to the slow sound velocity compared to that of the light, the SOI effect of phonons can be observed at relatively slow rotating rate (<1 Hz), whereas the Fizeau drag of light was measured at several kHz ultrafast spinning speed.

## Discussion

In conclusion, we have established a PnIC architecture based on GaN-on-sapphire semiconductor substrates. Low loss single-mode waveguides, chip-to-cable connectors, evanescent directional couplers, and waveguide coupled high-*Q* acoustic ring resonators have been presented to demonstrate basic functionalities of phononic circuits as an analog to PICs. A comparison between PnICs and PICs is shown in Table 2. The sensitivity and versatility of conventional acoustic-based sensing devices can be greatly enhanced by confining phonons in a small volume with long lifetime and routing propagating phonons in more complex circuits in the PnIC architecture. Improved performance of the circuit in a cryogenic environment paves the way for future interfacing with superconducting quantum devices. Additionally, our GNOS platform is compatible with PICs^40^, allowing the coupling of phonons and photons through optoacoustic interaction^53^ on a single chip. Therefore, quantum links between superconducting qubit and propagating optical field can be built based on the PnIC. Moreover, the ring geometry gives rise to the coupling between OAM and spin of phonons. This new phenomenon opens doors for applications such as acoustic gyroscopic and non-reciprocal devices.

**Table 2:** Comparison between the PnIC and the PIC

	PnIC	PIC
Input/Output	Interdigital transducer	Grating coupler
External ports	RF cable	Fiber
Interconnection	Phononic waveguide	Photonic waveguide
Velocity	4000 m s^−1^	1.3 × 10^8^ m s^−1^ (in GaN)
Modes	Rayleigh/Love-like	Quasi-TE/TM

## Methods

### Fabrication

A detailed description of fabrication procedures is provided in the Supplementary Note 2. In brief, our PnIC devices are patterned by electron-beam lithography and dry etching processes on GaN-on-sapphire wafers with the GaN layer thickness of 5 μm or 10 μm. The IDTs are patterned by electron-beam lithography using polymethyl methacrylate (PMMA) resist, followed by the deposition of 10-nm-thick chromium and 50-nm-thick gold and a subsequent lift-off process in acetone.

### Measurement methods

A detailed description of measurement methods is provided in the Supplementary Note 2. In brief, the phononic circuit devices are characterized by two methods. The first is an optical measurement of the mechanical vibration of the sample surface by a home-built vibrometer with vibration amplitude sensitivity of around 70 fm Hz^−1/2^. The principle of the vibrometer is based on a quadrature measurement of vibration-modulated light signals by a heterodyne interferometer. The capability of simultaneous detection of amplitude and phase allows us to fully characterize the traveling acoustic modes in waveguides and ring resonators. In the frequency domain, it offers wide-range frequency spectrum. The second method is an electrical transmission measurement, by which the frequency spectrum *S*_21_ of the phononic device can be obtained conveniently using a network analyzer. This method is particularly suitable for the measurements in a cryogenic environment.

## Supplementary information

Supplementary information

## Data Availability

The data that support the findings of this study are available from the corresponding author (H.T.) upon reasonable request.
