# Supplementary material for: Phononic integrated circuitry and spin–orbit interaction of phonons
Source: Nat Commun. 2019 Jun 21;10:2743. doi: 10.1038/s41467-019-10852-3 (PMC6588612; doi:10.1038/s41467-019-10852-3)
Supplement: Supplementary file 1 — Supplementary information [file 41467_2019_10852_MOESM1_ESM.pdf]

Supplementary Information for 'Phononic integrated circuitry and spin-orbit interaction of phonons'

Fu et al.

SUPPLEMENTARY NOTE 1: THEORY AND SIMULATION

Waveguide and ring simulation

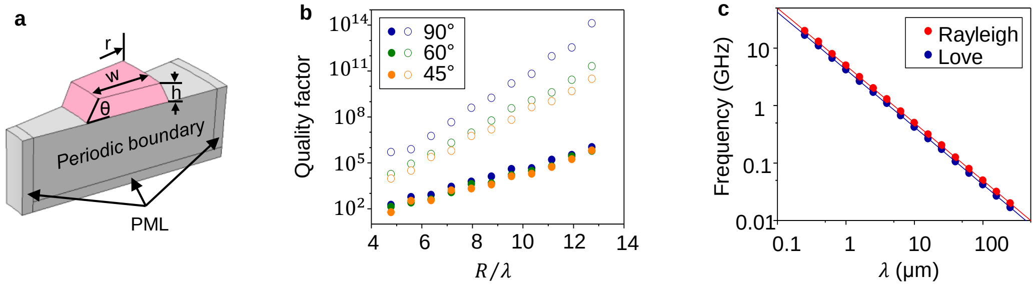

Supplementary Figure 1. **Simulation of ring resonator.** **a**, Geometry in the COMSOL Multiphysics simulation. Geometric parameters are defined as shown above. **b**, The quality factor (radiation Q) increases exponentially as radius of the ring increases. Solid dots represent Rayleigh-like modes, while circles represent Love-like modes. **c**, The inversely proportional relation between frequency and wavelength. μm wavelength design can scale the PnIC frequency up to the GHz range. PML: Perfectly matched layer.

We use a finite element method (COMSOL Multiphysics V5.2) to simulate and optimize the phononic waveguides and ring resonators. In Fig, 1**a**, without loss of generality, we present the geometry of a ring resonator in the simulation. We solve the eigenmodes in a section of the whole ring with periodic boundary condition $u(\theta) = u(0)\, e^{-im\theta}$, due to the cylindrical symmetry of the ring resonator, where u is the acoustic displacement, θ is the angle of the simulation section, and m is the azimuthal number of the whispering gallery mode (WGM). Perfectly matched layers are employed to absorb acoustic energy radiating away from the ring.

We first study the phonon modes in the phononic waveguide and ring resonator. By computing for the eigenmodes of waveguide and ring structure, we acquire phonon modes with different polorization as shown in Fig. 3**h** and Fig. 3**i**. Furthermore, we make a cut plane perpendicular to the propagation direction and calculate the total elastic energy $E_{\text{tol}} = E_{\text{kinetic}} + E_{\text{strain}}$, where E_{knetic} is the kinetic energy distribution and E_{strain} is the elastic strain energy distribution, to acquire the cross-sectional acoustic energy distribution shown in Fig. 1**c**.

We also study the radiation loss of ring by varying the radius of the ring and calculating the radiation-limited quality factor. Figure 1**b** indicates that the radiation-limited quality factors of both the Rayleigh-like and Love-like WGMs increase exponentially as radii become larger. Moreover, sidewall, which is introduced by fabrication imperfection, is taken into account by changing the sidewall angle from 90° to 45°. As shown in Fig. 1**b**, when the sidewall angle becomes smaller, both Rayleigh-like and Love-like mode have a lower radiation-limited quality factor. However, this issue can be easily compensated by designing the radius sufficiently large to eliminate the radiation loss.

To scale the circuit into GHz range, where phonon modes and microwave modes are immune to thermal noise in cryogenic conditions and can be easily prepared to their ground states, we numerically demonstrate the frequency of phonon as a function of the wavelength in GNOS, where material dispersion is negligible [1]. Figure 1**c** shows the inversely proportional relation between frequency and wavelength, with all the geometrical parameters scaled with the wavelength. In the GHz range, the wavelength is around μm, which can be easily implemented with a standard fabrication process. It is worth noting that at μm wavelength, which is comparable with the wavelength of 1550 nm photons in the GNOS platform, the frequency is in the $1 \sim 10$ GHz range, compatible with the frequency of common quantum electrical circuit, making the bridge between PIC and quantum electrical circuit based on PnIC possible.

In order to study the actuation of phonons, the directional coupler, and waveguide-ring coupler structure, we employ piezoelectric module and actuate phonon mode at the end of waveguides.

Phononic evanescent waveguide directional coupler

Multiport phononic devices are indispensable for a versatile circuit. For efficient connection between the components in the circuits, directional coupler structures are designed, simulated, and experimentally demonstrated in the form of wrap-around coupler. The coupler is implemented by closely placing two parallel waveguides (shown in inset of Fig. 2**a**), with their evanescent acoustic fields overlapping in the substrate. Consider the phonon modes in the two separate parallel waveguides, whose complex amplitudes are $\boldsymbol{A}$ and $\boldsymbol{B}$. The unperturbed eigenmodes of the two waveguides can be represented by

$$\boldsymbol{A}(\boldsymbol{r},t) = a(x)e^{i\omega t}\boldsymbol{u}_{\mathrm{A}}(y,z)\,, \tag{1}$$

$$\boldsymbol{B}(\boldsymbol{r},t) = b(x)e^{i\omega t}\boldsymbol{u}_{\mathrm{B}}(y,z)\,, \tag{2}$$

where x is the propagation direction, ω is the frequency acoustic wave, and $\boldsymbol{u}_{\mathrm{A,B}}(y,z)$ is the displacement field of acoustic eigenmode at the cross-section of waveguide A and B.

When the two waveguides are close to each other, the phonon can tunnel between them due to the mode overlap. For two identical waveguides, the evolution of mode amplitudes a and b along the propagation direction obeys

$$\frac{d}{dx}a = -ika - h_{\mathrm{ab}}b, \tag{3}$$

$$\frac{d}{dx}b = -ikb - h_{\mathrm{ba}}a, \tag{4}$$

where k is the wavenumber of the mode, $h_{\mathrm{ab(ba)}}$ is the coupling rate. Considering energy conservation, $h_{\mathrm{ab}} = -h^*_{\mathrm{ba}}$. We can solve the eigenvalue of the equations above to be $k_\pm = k \pm h$, where $h \equiv |h_{\mathrm{ab}}|$ is the coupling strength, and the normal modes of the coupled system are $a+b$ (symmetric mode) and $a-b$ (anti-symmetric mode). For a directional coupler, we have $a(0) = 1$ and $b(0) = 0$, and we can get

$$a(x) = \cos(hx)e^{-ikx}, \tag{5}$$

$$b(x) = \sin(hx)e^{-ikx}, \tag{6}$$

where phonons hop between two coupled waveguides. In the simulation, with fixed wavenumber for acoustic mode, different eigenfrequencies $\omega_\pm = \omega \pm vh$ are observed for symmetric and anti-symmetric modes and coupling strength can be calculated accordingly by equation $h = {}^{(\omega_+ - \omega_-)}/_{2v}$, where v is the phase velocity of acoustic wave.

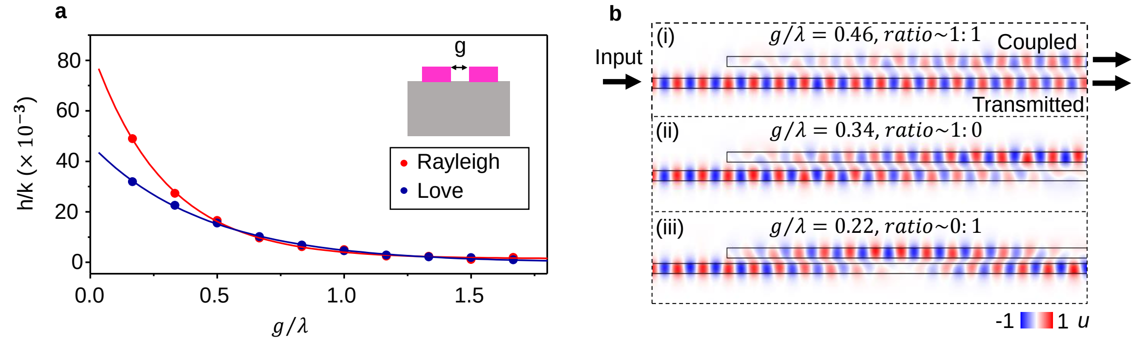

Supplementary Figure 2. **Evanescent phononic directional coupler.** **a**, Numerically simulated dependence of coupling strength h on gap g between two waveguides. Rayleigh-like and Love-like are indicated by red and blue dots, respectively, fitted with exponential function. **b**, Simulation results of phononic directional coupler. As the coupling gap becomes larger, the coupling is weaker, leading to different power splitting ratios between coupled and transmitted ports. In this simulation, the ratios are approximately: (i) 1 : 1, (ii) 1 : 0 (iii) 0 : 1.

Figure 2**a** presents the simulation result of coupling strength. Due to the exponentially decaying evanescent wave, the coupling coefficient h between the waveguides drops correspondingly with the increase of the gap (g). With such engineerable coupling coefficient, the ratio of phonon coupled to the other waveguide can be accurately estimated as $\sin^2(hL)$, where L is the length of the coupling region. Therefore, the multiport phononic devices, such as Y-junction,

power splitter, Mach-Zehnder interferometer, and wrap-around coupler, can be achieved based on such design. As a proof of concept, a simulation of phononic directional coupler in Fig. 2**b** is conducted, where the transmission ratio between the coupled port and transmitted port is adjusted by changing the gap. Within a coupling length of approximately 10-wavelengths, the transmission ratio can be freely tailored by engineering the gap in a range of $0.2\lambda \sim 0.5\lambda$, which can be fabricated precisely by electron-beam lithography. With the theoretical framework of the evanescent directional coupler introduced, wrap-around couplers that enables the coupling between the phononic bus waveguide and ring resonator are experimentally realized and introduced in the main text.

Orbital angular momentum, spin, and chirality

Two important concepts underpin the SOI of phonon: orbital angular momentum and spin. Propagating phonons carry momentum $\boldsymbol{P} = \hbar\boldsymbol{k}$. For phonons circulating in phononic rings, we define orbital angular momentum $\boldsymbol{L} = \boldsymbol{r} \times \boldsymbol{P}$, where $\boldsymbol{r}$ is the position with respect to the center of the ring. Phonons that propagates towards clockwise (CW) and counter-clockwise (CCW) direction have orbital angular momentum pointing $-z$ and $+z$ direction, respectively. For love-like modes, we define spin as the circular-like motion of particles in the phononic rings. The local spins couple to orbital angular momentum and lead to the chirality of phonon mode. The chirality is defined as $\boldsymbol{\chi} = (\chi_{\mathrm{x}}, \chi_{\mathrm{y}}, \chi_{\mathrm{z}})$, and $\chi_i = -i \iiint_V dx\, dy\, dz\, \epsilon_{ijk} u_j^* u_k$ [2], where u_j is normalized displacement along j direction and ϵ_{ijk} is the Levi-Civita symbol. For instance, an ideal CW (CCW) rotating Foucault pendulum has $\chi_{\mathrm{x}} = \chi_{\mathrm{y}} = 0$ and $\chi_{\mathrm{z}} = -1(1)$. For a circulating Love-like mode, the chirality is determined by the collective motion of all the particles in the phononic ring. From simulation results, we calculate the chirality of both CW and CCW modes, $\chi_{\mathrm{x,cw}} = \chi_{\mathrm{x,ccw}} = \chi_{\mathrm{y,cw}} = \chi_{\mathrm{y,ccw}} = 0$, while $\chi_{\mathrm{z,cw}} = -\chi_{\mathrm{z,ccw}} = -0.12$. It is worth noting that $\boldsymbol{\chi} \cdot \boldsymbol{L} > 0$, owing to the SOI.

Gyroscopic effect and non-reciprocal phase shift

The gyroscopic effect of phonon spin is induced by Coriolis force. Without rotation, the motion of a particle with circular-like trajectory can be described by $x = a\cos\omega_0 t$, $y = b\sin\omega_0 t$, representing an elliptic motion in the $x-y$ plane with respect to the rotation along z-direction ($\boldsymbol{e_z}$). When the particle is subject to rotation in z-direction with $\Omega_{\mathrm{z}} > 0$, the Coriolis force on the particle is $\boldsymbol{F} = -2m\Omega\boldsymbol{e_z} \times \boldsymbol{v}$. Let $x \pm iy = A_\pm e^{-i\omega_\pm t}$, where A_+ (A_-) and ω_+ (ω_-) represent the amplitude and frequency of CW (CCW) rotation, the equation of motion can be written as

$$-A_\pm\omega_\pm^2 + A_\pm\omega_0^2 = \mp 2\Omega A_\pm\omega_\pm, \tag{7}$$

For $\Omega \ll \omega_0$, we have

$$\omega_\pm = \omega_0 \pm \Omega. \tag{8}$$

Therefore, the Coriolis force of acoustic wave leads to non-reciprocal frequency shift between two spin modes.

In the phononic ring resonator, CW and CCW phonons possess opposite chirality, thus have non-reciprocal response to the rotation in z-direction. Their resonant frequency become [2, 3]:

$$\omega = \omega_0 - \boldsymbol{\chi} \cdot \boldsymbol{\Omega}, \tag{9}$$

where we $\boldsymbol{\chi}$ is the chirality of the phonon we defined in the previous section.

We consider the equations of motion with only CW input,

$$\begin{aligned}\frac{\mathrm{d}}{\mathrm{dt}}\begin{pmatrix} a_{\mathrm{cw}} \\ a_{\mathrm{ccw}} \end{pmatrix} &= \begin{pmatrix} \chi_0 - i\boldsymbol{\chi}\cdot\boldsymbol{\Omega} & -ih \\ -ih & \chi_0 + i\boldsymbol{\chi}\cdot\boldsymbol{\Omega} \end{pmatrix}\begin{pmatrix} a_{\mathrm{cw}} \\ a_{\mathrm{ccw}} \end{pmatrix} \\ &\quad - \begin{pmatrix} \sqrt{2\kappa_{\mathrm{ex}}} & 0 \\ 0 & \sqrt{2\kappa_{\mathrm{ex}}} \end{pmatrix}\begin{pmatrix} a_{\mathrm{cw}}^{\mathrm{in}} \\ 0 \end{pmatrix}.\end{aligned} \tag{10}$$

where a_{cw} (a_{ccw}) is annihilation operator for CW (CCW) mode, $\chi_0 = -i(\omega - \omega_0) - \kappa$. ω and ω_0 are the frequency of input signal and original resonant frequency, respectively. κ and κ_{ex} are total cavity decay rate and external coupling strength, respectively. h is the backscattering induced coupling between CW and CCW modes. The output of the system can be described by

$$\begin{pmatrix} a_{\mathrm{cw}}^{\mathrm{out}} \\ a_{\mathrm{ccw}}^{\mathrm{out}} \end{pmatrix} = \begin{pmatrix} a_{\mathrm{cw}}^{\mathrm{in}} \\ 0 \end{pmatrix} + \begin{pmatrix} \sqrt{2\kappa_{\mathrm{ex}}} & 0 \\ 0 & \sqrt{2\kappa_{\mathrm{ex}}} \end{pmatrix}\begin{pmatrix} a_{\mathrm{cw}} \\ a_{\mathrm{ccw}} \end{pmatrix}. \tag{11}$$

The solution for CW output is

$$\frac{a_{\rm cw}^{\rm out}}{a_{\rm cw}^{\rm in}} = 1 + \frac{2\kappa_{\rm ex}\left(i\boldsymbol{\chi}\cdot\boldsymbol{\Omega}+\chi_0\right)}{(\boldsymbol{\chi}\cdot\boldsymbol{\Omega})^2+\chi_0^2+h^2} \tag{12}$$

When $\boldsymbol{\chi}\cdot\boldsymbol{\Omega}\ll\kappa$, the phase shift of the output can be approximated as

$$\phi_{\rm cw} \approx \boldsymbol{\chi}\cdot\boldsymbol{\Omega}\frac{\partial}{\partial\omega_{\rm cw}}\arg\left(a_{\rm cw}^{\rm out}\right). \tag{13}$$

We measure the phase response of CW and CCW simultaneously, and obtain the difference of phase shift

$$2\Delta\phi = \phi_{\rm cw}-\phi_{\rm ccw} \approx \boldsymbol{\chi}_{\rm cw}\cdot\boldsymbol{\Omega}\frac{\partial}{\partial\omega_{\rm cw}}\arg\left(a_{\rm cw}^{\rm out}\right) - \boldsymbol{\chi}_{\rm ccw}\cdot\boldsymbol{\Omega}\frac{\partial}{\partial\omega_{\rm ccw}}\arg\left(a_{\rm ccw}^{\rm out}\right) \approx \frac{\boldsymbol{\chi}_{\rm cw}\cdot\boldsymbol{\Omega}}{\pi}\frac{\partial}{\partial f_{\boldsymbol{0}}}\arg\left(a_{\rm cw}^{\rm out}\right), \tag{14}$$

where $a_{\rm ccw}$ is excited by driving tune $\omega_{\rm ccw}\neq\omega_{\rm cw}$ and $|\omega_{\rm ccw}-\omega_{\rm cw}|\ll\kappa$. The non-reciprocal phase shifts are added constructively while the noises (such as temperature induced drifts) are suppressed.

SUPPLEMENTARY NOTE 2: EXPERIMENT

Fabrication of phononic circuits

The fabrication of PnIC chip starts with the growth of an epitaxial GaN film over a *c*-plane sapphire substrate wafer using metal-organic chemical vapor deposition (MOCVD) [4]. We first define the phononic structures such as waveguides and ring resonators on Hydrogen silsesquioxane (HSQ) resist by electron-beam lithography (EBL). Then Chlorine-based reactive ion-etching (RIE) is employed to transfer the pattern to the GaN film layer. To define the electronic structures such as interdigital transducers (IDTs) or electronic shield, a second EBL is used to pattern the polymethyl methacrylate (PMMA) resist, followed by a deposition of 10-nm-thick chromium and 50-nm-thick gold by thermal evaporation and a lift-off process.

Vibrometer setup

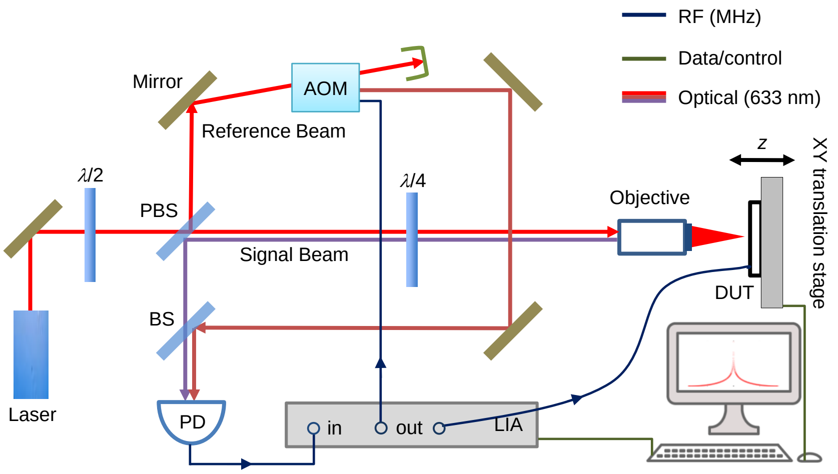

Supplementary Figure 3. **Experimental setup of the optical vibrometer.** Schematic of the vibrometer setup. AOM: acousto-optic modulator. PBS: polarizing beam splitter. $\lambda/2$: half-wave plate. $\lambda/4$: quater-wave plate. BS: beam splitter. LIA: lock-in amplifier. PD: photodetector. DUT: device under test. RF: radio frequency signal.

Figure 3 shows the schematic illustration of the home-made vibrometer setup used in our experiments. A linearly polarized 633nm HeNe laser is split into two paths, the reference beam and the signal beam. The half-wave plate

($\lambda/2$) is used to tune the power ratio between the reference and signal beams. The frequency of the reference beam is shifted from the original input laser frequency ω to $\omega + \omega_{\text{ref}}$ by an acousto-optic modulator (AOM). The signal beam is normally focused on the sample with a microscope objective (NA = 0.55) and reflected back to the PBS. The polarization of the reflected probe signal is rotated by 90 degrees because of the double pass through the $\lambda/4$ plate, and then the signal is reflected by the PBS and guided to the detector. Thus, the frequency-shifted reference light and the sample motion-modulated signal have the same polarization and they interfere on the photodetector (PD). The signal outputs of the lock-in amplifier (Zurich instruments UHFLI) are amplified to drive the AOM and surface acoustic wave (SAW) device. The device is placed on a linear piezo stage (Micronix PPS-20) with lateral resolution of 10 nm, which allows two-dimensional point-by-point scanning imaging.

For a brief description of the heterodyne detection, we assume the sample surface vibrates sinusoidally as $Z_0\sin(\Omega t+\phi)$ in the z-direction, where Z_0, Ω and ϕ are the amplitude, angular frequency and phase of the vibration, respectively. The signal light field detected by PD is $E_{\text{sig}} = A_1\cos[\omega t + \phi_1 + 2kZ_0\cos(\Omega t + \phi)]$ and the reference light field is $E_{\text{ref}} = A_2\cos[(\omega + \omega_{\text{ref}})t + \phi_2]$, where A_1 (A_2) and ϕ_1 (ϕ_2) denote the amplitudes and phases of the signal (reference) field, and $k = 2\pi/\lambda$ is the optical wavenumber. The PD's response to the intensity of light field is $I(t) \approx |E_{\text{sig}} + E_{\text{ref}}|^2 = A_1A_2\cos(\omega_{\text{ref}}t + \phi_2 - \phi_1) + A_1A_2kZ_0\cos[(\Omega - \omega_{\text{ref}})t + \phi + \phi_1 - \phi_2)]$. Here, we drop the second-order term of Z_0^2 and also filter the component oscillating at high frequency $\Omega + \omega_{\text{ref}}$. When the lock-in amplifier demodulates the photodetected signal at $\omega_{\text{ref}}/2\pi$ and $(\Omega - \omega_{\text{ref}})/2\pi$ simultaneously, the amplitude $R_{\text{ref}} = A_1A_2$ ($R_{\text{sig}} = kZ_0R_{\text{ref}}$) and phase $\theta_{\text{ref}} = \phi_2 - \phi_1$ ($\theta_{\text{sig}} = \phi + \phi_1 - \phi_2$) of the reference (signal) peak are determined. Finally, the vibration amplitude and phase of surface can be calculated as $Z_0 = \frac{1}{k}\frac{R_{\text{sig}}}{R_{\text{ref}}}$, and $\phi = \theta_{\text{sig}} + \theta_{\text{ref}}$. The vibration amplitude sensitivity of the vibrometer is around $70\,\text{fm}\,\text{Hz}^{-1/2}$, which is mainly limited by electrical noise of the PD.

Optical and electrical readout of acoustic devices

To assess the SAW modes in the waveguides and rings, we use the vibrometer to record both amplitude and phase information at a fixed excitation frequency and RF power. The two-dimensional displacement distribution ($Z_0\sin(\Omega t + \phi)$ at a fixed t) of the device is constructed from the experimental data, as shown in Figs. 2 and 3 of the main text.

The optically transduced displacement spectra of phononic ring resonators shown in the main text are obtained adjusting the laser focal point at a fixed point on the ring while the the excitation frequency is swept at a constant RMS amplitude of 100 mV. The electrical transmission (S_{21}) measurement is carried out using a network analyzer connected to a pair of IDTs on the device.

Intrinsic and external decay rate of phononic ring resonator

To understand the phonon loss mechanisms and optimize the performance of the PnIC, we study the resonances around 200 MHz ($\lambda = 25\,\mu$m) to determine how linewidth depends on the geometric parameters and environmental temperature. Phononic circuit devices, as shown in Fig. 4**a**, consist of rings with varied geometries. The arrays of rings can be excited simultaneously through a single evanescently-coupled waveguide while measured individually by adjusting the laser focal point on each ring. A typical resonant spectrum fitted with the Lorentzian function is shown in Fig. 4**b**. The linewidth of each ring can be extracted from the fitting and is determined by the average of 4 resonances around 200 MHz. The resonance presented in Fig. 4**b** has linewidth of 8 kHz, which gives a lower limit of quality factor of 2.5×10^4 and phonon lifetime of $20\,\mu$s at room temperature.

There are several factors that contribute to the measured linewidth, including the external decay rate (κ_{ex}) and intrinsic decay rate (κ_{in}). The κ_{ex} is introduced by the wrap-around coupling of the ring and the waveguide as introduced in the previous section. As shown in Fig. 4**c**, the experimental coupling coefficient drops exponentially when gap (g) increases. The intrinsic loss rate κ_{in} includes the radiation loss (κ_{rad}) due to the waveguide bending and the material limited loss (κ_{mat}). In order to determine the dependence of κ_{rad} on the radius of curvature, we sweep the radii of the rings with g fixed to be $14\,\mu$m ($g/\lambda = 0.56$). Figure 4**d** shows a rapid decrease of linewidth when the radius increases, indicating that the radiation loss of the ring has an exponential dependence on radius (supported by simulation in previous section). These experimental results show that the linewidth narrows when the geometry (increasing g and R) are optimized and saturates to a certain value limited by the intrinsic material loss, which, according to previous studies of the Akhiezer effect [5], is introduced by quantum mechanical phonon processes and can be suppressed in a cryogenic environment.

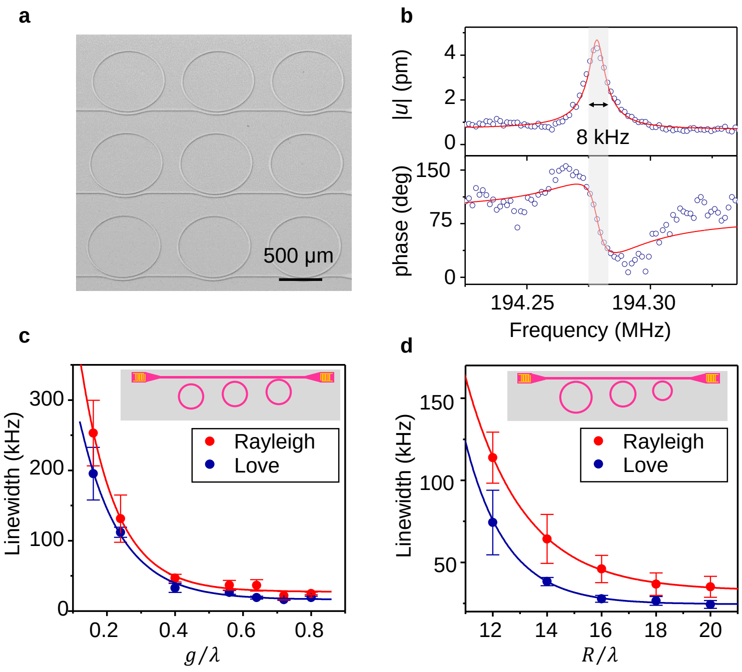

Supplementary Figure 4. **Loss mechanisms of phononic circuits**. **a**, SEM of the phononic circuit. Each waveguide couples to many ring resonators with varied geometries. **b**, A resonant amplitude and phase spectrum measured by the vibrometer, fitted with the Lorentzian function. **c**, The linewidth of phononic WGMs against the gap. **d**, The linewidth of phononic WGMs against the radius. All data in (**c**) and (**d**) are fitted by exponential functions, which is supported by numerical results (in 'Waveguide and ring simulation' section). Error bars represent standard deviation.

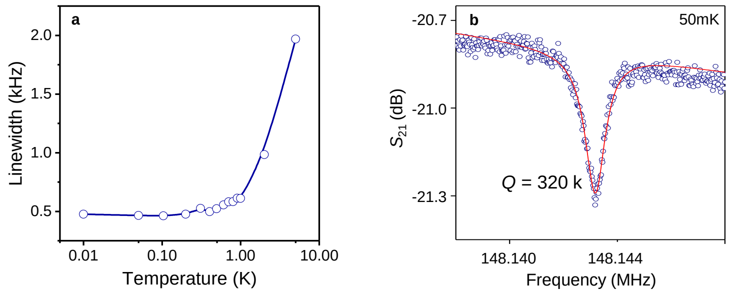

Supplementary Figure 5. **Cryogenic measurement.** **a**, Dependence of linewidth of a resonance on the temperature. Blue line in the figure is a guide to the eye. **b**, Resonance with the miximum Q is measured at 50 mK.

Material loss at cryogenic environment

A device fabricated from 10 μm-thick GaN to support phonon modes with wavelength of around 30 μm is loaded in a dilution refrigerator (Bluefors), which provides a cryogenic environment (10 mK – 8 K). Figure 5**a** shows the linewidth of a resonance at varied temperature. As the temperature goes down, the material loss decreases dramatically [5]. At 50 mK temperature, the highest Q of 3.2×10^5 is observed. With material loss greatly reduced, the performance of the ring resonators is once again limited by κ_{rad} or κ_{ex}, which can be further diminished by engineering the geometric parameters and improving the fabrication processes. Therefore, we are able to place a lower limit on the achievable quality factor of our GONS phononic platform: $Q \geq 3.2 \times 10^5$ at 150 MHz and 50 mK, corresponding to the achievable phonon lifetime $\tau \geq 0.34$ ms.

Spin-orbit interaction measurement

To study the non-reciprocal phase response of CW and CCW Love modes, we employ a experimental setup as shown in Fig. 6**a** . The setup comprises four major blocks: a device under test (DUT), a RF signal conditioning circuit, a lock-in amplifier and a Microsoft surface computer. Blue and red lines are transmission lines for CW and CCW signal, respectively. Purple lines represent the Aluminum wire bonds connecting the transmission lines and IDTs. Universal serial bus (USB) controls are indicated by green lines. By employing two power splitters, CW and CCW signaling paths share the same SAW components and wire bonds, which is crucial for us to cancel unwanted drift in the device caused by environment, such as thermal effect or vibration of the bonded aluminum wire. Also, the symmetric RF conditioning circuit inside the black dash line is sealed in a box to further suppress the environmental fluctuation. We use a lock-in amplifier (Zurich Instruments UHFLI) as well as a computer (Microsoft surface) to collect data and control the system. Within the lock-in amplifier, two oscillators are used to generate and demodulate CW and CCW signals, and phases of CW and CCW signal are detected simultaneously.

To achieve better signal to noise ratio, the design of the IDTs are optimized by employing a focusing structure to improve the conversion efficiency to $-25\,\mathrm{dB}$ [6]. The electrode shields around the IDTs are introduced to suppress the cross-talk between the two IDTs. For further improvement of the electromechanical conversion efficiency, IDTs with larger areas or phononic Fabry-Parot cavities [7, 8] can be used to realize the impedance matching condition. As shown in Fig. 6**b** is the transmission spectrum of the DUT. A resonance at $117.43\,\mathrm{MHz}$ (mode a) is chosen for its relative high Q (2.0×10^4) and large extinction ($\sim 5\,\mathrm{dB}$). By fitting the phase response of the transmission, we acquire the slope of the resonance be $\frac{\partial}{\partial f_{\mathrm{cw}}}\arg\left(a_{\mathrm{cw}}^{\mathrm{out}}\right) \approx \frac{\partial}{\partial f_{\mathrm{ccw}}}\arg\left(a_{\mathrm{ccw}}^{\mathrm{out}}\right) = 0.0064\,\mathrm{deg\,Hz^{-1}}$, which is used to determine the $\boldsymbol{\chi}$ in the main text. Moreover, to cancel the fluctuation and drift in electrical conditioning circuit, we use two off-resonant reference signals that share the exact same electric path with the CW and CCW probe signals, respectively. The four signals are indicated by correspondingly colored circles in Fig. 6**c**, and frequencies of CW and CCW probes and references swap every $10\,\mathrm{ms}$ so as to make the system more symmetrical. A phase lock loop (PLL) inside the UHFLI is employed and locked to the adjacent Love-like mode (mode b) to track the real-time resonant frequency, which drifts due to the temperature fluctuation. The whole measurement setup is mounted on a close-loop controlled rate table (Acutronic AC1120S) .

Supplementary References

[1] Ichihashi, H., Yanagitani, T., Takayanagi, S., Kawabe, M. & Matsukawa, M. Gigahertz acoustic wave velocity measurement in gan single crystals considering acousto-electric effect. *IEEE Trans. Ultrason., Ferroelectr., Freq. Control* **61**, 1307–1313 (2014).

[2] Zou, C.-L., Xie, J., Fu, W., Jiang, L. & Tang, H. X. Spin-orbit coupling in a phononic resonator (In prep.).

[3] Lao, B. Y. Gyroscopic effect in surface acoustic waves. In *1980 Ultrasonics Symposium*, 687–691 (IEEE, 1980).

[4] Bruch, A. W. *et al.* Broadband nanophotonic waveguides and resonators based on epitaxial gan thin films. *Appl. Phys. Lett.* **107**, 141113 (2015).

[5] Ghaffari, S. *et al.* Quantum limit of quality factor in silicon micro and nano mechanical resonators. *Sci. Rep.* **3**, 3244 (2013).

[6] Vainsencher, A., Satzinger, K., Peairs, G. & Cleland, A. Bi-directional conversion between microwave and optical frequencies in a piezoelectric optomechanical device. *Appl. Phys. Lett.* **109**, 033107 (2016).

[7] Xu, Y., Fu, W., Zou, C.-L., Shen, Z. & Tang, H. X. High quality factor surface fabry-perot cavity of acoustic waves. *Appl. Phys. Lett.* **112**, 073505 (2018).

[8] Manenti, R. *et al.* Surface acoustic wave resonators in the quantum regime. *Phys. Rev. B* **93**, 041411 (2016). 1510.04965.

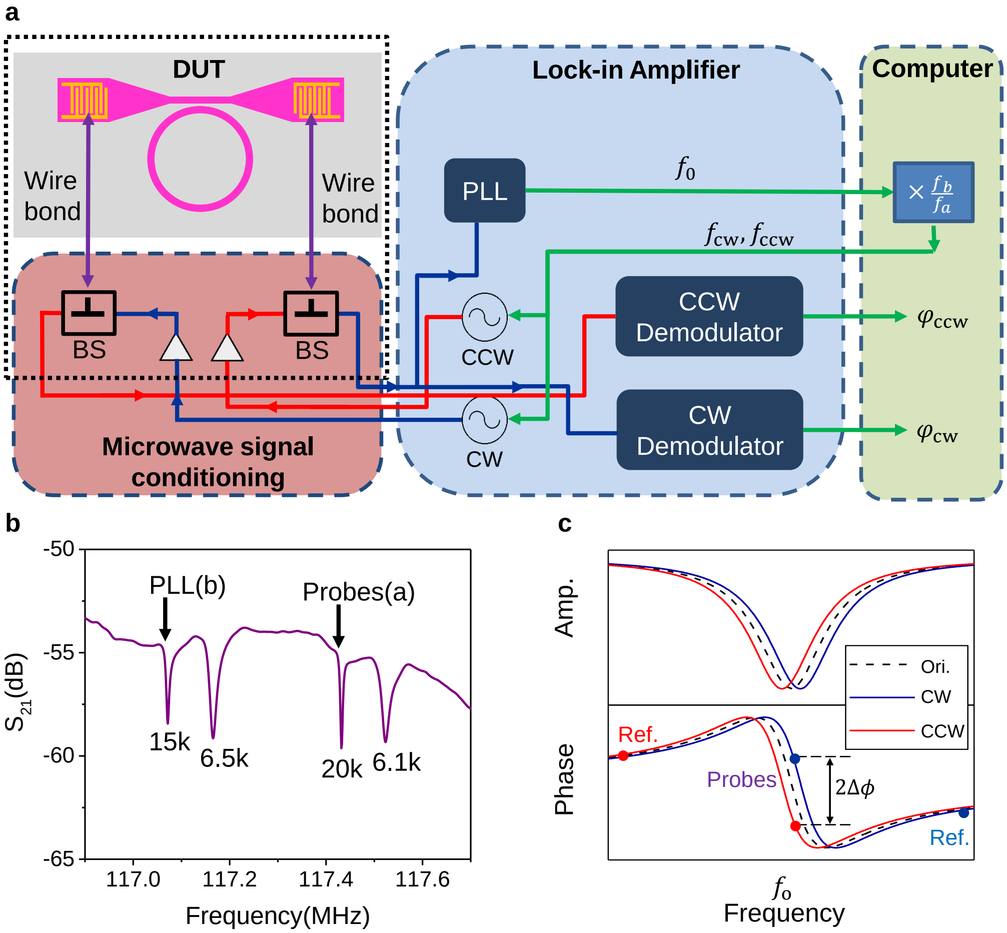

Supplementary Figure 6. **Spin-orbit interaction measurement.** **a**, Schematic of device and measurement setup. The blue (red) traces represent transmission lines for CW (CCW) signal. Purple lines are aluminum wire bonds shared by CW and CCW signals. Green traces are USB control. Arrows indicate the propagation direction of signals (or data). The DUT is an acoustic chip mounted on a PCB. The RF signal conditioning circuit includes transmission lines, amplifiers and power splitters. A PLL is used to track the frequency of resonance around 117.07 MHz, and then feedforward to adjust CW and CCW probe input frequencies to follow the target resonance. The parts in the black dashed line are sealed inside a box to further reduce the environment fluctuation, and are symmetric for CW and CCW signal. **b**, Transmission spectrum of the DUT. As labeled in the figure, PLL and probes are set on two Love modes in order to suppress environmental drifts and resonant frequency fluctuation. **c**, Illustration of phase change difference between CW and CCW signal $\Delta\phi$. CW and CCW probe and reference signals are indicated by blue and red dots, respectively. In the experiment, reference signals are farther detuned ($\Delta \gg \kappa$) from the resonance than as indicated in this figure. DUT: device under test. BS: beam splitter.
